# Plasma D-dimer levels are a biomarker for in-hospital complications and long-term mortality in patients with traumatic brain injury

**DOI:** 10.3389/fnmol.2023.1276726

**Published:** 2023-10-27

**Authors:** Xinli Chen, Xiaohua Wang, Yingchao Liu, Xiumei Guo, Fan Wu, Yushen Yang, Weipeng Hu, Feng Zheng, Hefan He

**Affiliations:** ^1^Department of Anesthesiology, The Second Affiliated Hospital of Fujian Medical University, Quanzhou, China; ^2^Department of Neurosurgery, The Second Affiliated Hospital of Fujian Medical University, Quanzhou, China

**Keywords:** traumatic brain injury, D-dimer, biomarker, in-hospital complications, long-term mortality, prognosis prediction

## Abstract

**Introduction:**

Traumatic brain injury (TBI) is a major health concern worldwide. D-dimer levels, commonly used in the diagnosis and treatment of neurological diseases, may be associated with adverse events in patients with TBI. However, the relationship between D-dimer levels, TBI-related in-hospital complications, and long-term mortality in patients with TBI has not been investigated. Here, examined whether elevated D-dimer levels facilitate the prediction of in-hospital complications and mortality in patients with TBI.

**Methods:**

Overall, 1,338 patients with TBI admitted to our institute between January 2016 and June 2022 were retrospectively examined. D-dimer levels were assessed within 24 h of admission, and propensity score matching was used to adjust for baseline characteristics.

**Results:**

Among the in-hospital complications, high D-dimer levels were associated with electrolyte metabolism disorders, pulmonary infections, and intensive care unit admission (*p* < 0.05). Compared with patients with low (0.00–1.54 mg/L) D-dimer levels, the odds of long-term mortality were significantly higher in all other patients, including those with D-dimer levels between 1.55 mg/L and 6.35 mg/L (adjusted hazard ratio [aHR] 1.655, 95% CI 0.9632.843), 6.36 mg/L and 19.99 mg/L (aHR 2.38, 95% CI 1.416–4.000), and >20 mg/L (aHR 3.635, 95% CI 2.195–6.018; *p* < 0.001). D-dimer levels were positively correlated with the risk of death when the D-dimer level reached 6.82 mg/L.

**Conclusion:**

Overall, elevated D-dimer levels at admission were associated with adverse outcomes and may predict poor prognosis in patients with TBI. Our findings will aid in the acute diagnosis, classification, and management of TBI.

## 1. Introduction

Traumatic brain injury (TBI) is a major health concern worldwide, affecting an estimated 54–60 million people annually. Moreover, according to the European Union, TBI contributes to 37% of all injury-related deaths worldwide (Vos et al., 2016; Maas et al., 2022). TBI is a systemic disease that can lead to organ dysfunction, including infections, respiratory and renal failure, myocardial injury, and multiple organ dysfunction in the acute phase, thereby increasing the length of hospital stays and costs (Stocchetti and Zanier, 2016). After discharge, many patients with TBI require long-term rehabilitation to address physical disability, cognitive impairment, and psychological disorders, which impose considerable economic burdens on families and society (Wiles et al., 2023). In addition, the poor prognosis of TBI substantially impacts patients, families, and healthcare providers. Therefore, early assessment of TBI is crucial for identifying critically ill patients, guiding treatment decisions, and establishing realistic expectations.

In clinical management, repeated computed tomography (CT) and the Glasgow Coma Scale (GCS) score aid in the evaluation of TBI. However, CT scans expose patients to ionizing radiation, and the potential long-term effects on children and pregnant women remain unclear. Furthermore, patients with severe TBI face risks during transportation and CT examinations (Kamiya et al., 2015; Riemann et al., 2023). The GCS score, determined based on the patient’s symptoms and the evaluator’s judgment, does not provide an accurate reflection of the extent of brain injury (Maas et al., 2017). Thus, at present, the commonly used diagnostic and classification methods are insufficient for all TBI groups. A convenient and sensitive objective index that reflects injury severity is required to supplement the diagnosis and prognosis of TBI (Maas et al., 2022; Nelson et al., 2023; Riemann et al., 2023).

Nearly two-thirds of patients with severe TBI have abnormalities on coagulation tests at admission (Maegele et al., 2017). Prior studies have shown that coagulation disorders strongly predict TBI prognosis, and coagulation disorders increase mortality risk and adverse outcomes by nine and 30 times, respectively, in patients with TBI (Greuters et al., 2011; Maegele et al., 2017). Coagulation dysfunction after TBI may be secondary to tissue damage, hypotension, and upregulation of inflammation, leading to an imbalance of coagulation and fibrinolysis pathways (Anderson et al., 2021). The coagulation system, which forms a thrombus to stop bleeding, and the fibrinolytic system, which dissolves a thrombus following tissue repair, work in harmony to establish hemostasis mechanisms. However, because of the exhaustion of coagulation factors and amplification of the fibrinolytic system in cases of severe trauma, the fibrinolytic system tends to become relatively overactive. This could lead to catastrophic and progressing coagulopathy (Maegele et al., 2017). After TBI, coagulation and fibrinolysis imbalances lead to a bleeding tendency, which increases the risk of progressive bleeding injury and intracranial hemorrhage and worsens the prognosis (Folkerson et al., 2015).

Cross-linked fibrin produces D-dimers. In a study of TBI-induced coagulation process changes, D-dimer levels reacted rapidly and increased within a few minutes of TBI (Zhang et al., 2018). Studies have found that brain-derived cellular microvesicles, a type of microvesicle that is rich in tissue factors and phosphatidylserine, are rapidly released into the circulation through the damaged blood–brain barrier after TBI, inducing systemic hypercoagulability and rapidly developing into consumptive coagulation dysfunction (Tian et al., 2015). This change is consistent with the time shift in which D-dimer peaks rapidly after TBI, while prolonged prothrombin time and partial prothrombin kinase peak later (Zhang et al., 2018). According to previous studies, patients with brain parenchymal damage show a significant correlation between serum D-dimer levels and tissue factor levels (Suehiro et al., 2019). Compared to other tissues, the brain parenchyma has a higher tissue factor per unit weight. This increased tissue factor per unit weight makes the brain parenchyma more susceptible to conspicuous coagulation and the related hyperfibrinolysis, especially in cases of severe head trauma (Echigo et al., 2017). We speculate that the brain-derived cellular microvesicles and tissue factor may be the reason for the early increase in D-dimer in TBI.

D-dimer is commonly used to diagnose and treat neurological diseases because it is inexpensive and convenient. Elevated levels of D-dimer have been observed in the peripheral blood of patients with central nervous system diseases such as aneurysmal subarachnoid hemorrhage (Fang et al., 2022) and stroke (Qiu et al., 2023), indicating activation of systemic coagulation. Furthermore, D-dimer assessment is also useful for evaluating venous thrombosis, pulmonary embolism, and early diffuse intravascular coagulation following TBI (Wada et al., 2017; Chen et al., 2023). Therefore, it is worth investigating whether D-dimer levels are associated with adverse events during hospitalization and long-term prognosis after discharge in patients with TBI.

Recent studies have linked hyperfibrinolysis to tissue damage and trauma severity in patients with and without TBI (Hayakawa et al., 2017). Among patients with TBI, those with elevated peripheral blood D-dimer levels were found to be at risk of progressive hemorrhagic injury (Xu et al., 2020). Furthermore, D-dimer levels were found to predict a poor neurological prognosis 6 months after discharge in patients with isolated TBI (Fujiwara et al., 2022). Additionally, measurement of the D-dimer level can reduce unnecessary CT scans for head trauma assessment in children with TBI (Berger et al., 2015; Langness et al., 2018). Understanding the prognostic factors aids in TBI severity assessment and treatment planning. However, the relationship between D-dimer levels, TBI in-hospital complications, and long-term mortality in patients with TBI have not yet been investigated. These relationships should be studied to improve the diagnosis and treatment of brain trauma. Here, we hypothesized that D-dimer levels are associated with in-hospital complication rates and long-term mortality in patients. This large single-center cohort study examined the relationship between D-dimer levels, TBI complications, and mortality rates. This is the first study to explore this association.

## 2. Materials and methods

### 2.1. Study design

In this retrospective, single-center cohort study, patient data were obtained from the electronic medical records of patients admitted to the Second Affiliated Hospital of Fujian Medical University between January 2016 and June 2022. Clinical features and results were obtained from the electronic medical records and reviewed by a trained team of physicians. The data collected included details on sex, age, pre-existing medical conditions, clinical test results, clinical diagnosis and treatment, complications, and long-term follow-up.

This study adhered to the tenets of the Declaration of Helsinki and was approved by the Ethics Committee of the Second Affiliated Hospital of Fujian Medical University (2022, No. 523). Each patient or their authorized representative was verbally informed about the purpose of the study and the potential risks of participating at the time of admission and agreed to an anonymous analysis of personal data for scientific purposes. Referring to other retrospective study designs, the requirement for informed consent can be waived for anonymous data (Filion et al., 2016). As a supplement to the informed consent procedure, the patients or their authorized signed the informed consent form at the last follow-up of each patient.

### 2.2. Patient selection

The inclusion criteria for this study were as follows: (1) patients diagnosed with TBI by neurosurgeons based on neuroimaging and managed in accordance with the “Guidelines for the Management of Severe Traumatic Brain Injury” and (2) patients without severe trauma to other organs.

The exclusion criteria were as follows: (1) patients with a history of coagulation disorders; (2) patients who received anticoagulant therapy or blood replacement therapy that may lead to coagulopathy before admission; (3) patients with diseases that affect D-dimer levels, such as malignant tumors, uremia, cirrhosis, and immune system diseases; (4) lack of D-dimer test results within 24 h of admission; (5) incomplete medical records; and (6) loss to follow-up.

### 2.3. D-dimer measurement

For this study, the exposure variable was the concentration of D-dimer in the blood. Blood samples were collected from patients with TBI within 24 h of admission. The patients were divided into four groups according to their D-dimer levels. As the upper limit of the detection machine was 20 mg/L, the highest D-dimer level group had D-dimer levels higher than 20 mg/L; patients with D-dimer levels below 20 mg/L were divided into three groups according to the percentile method. Finally, the baseline cut points of the four groups were 0.00–1.54 (n = 326), 1.55–6.35 (n = 325), 6.36–19.99 (n = 324), and ≥ 20.00 mg/L (n = 363).

### 2.4. Outcome measures

The primary outcome was long-term mortality (defined as mortality at the longest follow-up). Secondary outcomes were 1-year mortality (defined as mortality at the 1-year follow-up), 1-month mortality (defined as mortality at the 1-month follow-up), and in-hospital complications. In-hospital complications included serum electrolyte disorder, hypoproteinemia, cardiac complications (including cardiac insufficiency, arrhythmia, myocardial damage), urinary complications (renal insufficiency, urinary tract infection, diabetes insipidus), traumatic coagulopathy, pulmonary infection, admission to the intensive care unit (ICU), gastrointestinal complications (gastrointestinal dysfunction, gastrointestinal infection, gastrointestinal ulcer, gastrointestinal perforation, gastrointestinal bleeding), hypohepatia, stress hyperglycemia, deep vein thrombosis, seizures, and intracranial infection.

### 2.5. Follow-up

Patients were discharged between January 1, 2016, and May 31, 2022, and the follow-up ended on December 31, 2022. The average follow-up time was 2.8 years, ranging from 0.5 to 6.9 years. According to the follow-up guidelines for discharged patients in our research center, each patient was followed up by a doctor at 1 month, 3 months, 6 months, and 1 year after discharge, and their health status was recorded; the surviving patients who had been discharged for more than 1 year were followed up at least once a year. If the patient died, the follow-up was stopped and the date of death was recorded.

### 2.6. Data collection and clinical assessment

Medical records of patients with TBI were retrospectively analyzed. We extracted the following information: demographic data (age and sex), medical history (hypertension and diabetes), Glasgow Coma Scale (GCS) score at admission, and whether surgery was performed in this hospital. Laboratory test data were also collected within 24 h of admission, including the D-dimer concentration, hemoglobin (HB) concentration, platelet (Plt) count, fibrinogen (Fib) concentration, activated partial thromboplastin time (APTT), thrombin time (TT), and prothrombin time (PT). We also recorded the occurrence of complications at the hospital.

### 2.7. Statistical analysis

R version 4.0.3 (R Core Team, 2020) and IBM SPSS 26 (IBM Corporation, 2019) were used for all statistical analyses. Continuous baseline data are presented as mean with the standard deviation and were compared using analysis of variance. Chi-square tests were used to compare the counts (frequencies) of categorical variables. *p* < 0.05 was considered to indicate statistical significance in all two-sided significance tests. For categorical variables, all missing values were encoded as other variables, and multiple inferences were used for continuous variables.

Propensity score matching (PSM) reduced the bias of confounding variables (age, GCS score, and surgical treatment) and other important biomarkers (HB concentration, Plt count, fibrinogen concentration, APTT, TT, and PT). The similar groups were matched 1:1, and the matching tolerance was 0.20 SD. D-dimer levels and patient factors determined that a logistic regression score > 0.1 was significant. The PSM-adjusted dataset was subsequently analyzed.

The median method was used to divide D-dimer levels into high- and low-level groups to assess their relationship with in-hospital complications. A binary logistic regression model was used to identify the predictive outcome indicators. The multivariate logistic regression model included all univariate variables with bilateral *p* values <0.05 to determine the outcome variables associated with D-dimer levels.

Kaplan–Meier analysis was used to examine the unadjusted overall survival rate, while the log-rank test was used to determine significant differences between groups. Multivariate Cox regression analysis was used to examine D-dimer levels and mortality rates. Hazard ratios and 95% confidence intervals (CIs) are presented. The multivariate analysis incorporated significant (*p* < 0.05) univariate variables.

To investigate the dose–response relationship between death and D-dimer levels, we constructed a restricted cubic spline (RCS) curve. The cubic spline was adjusted for confounding factors to detect a possible nonlinear relationship between the D-dimer level as a continuous variable and long-term mortality.

D-dimer levels were assessed using receiver operating characteristic (ROC) curves to predict death at different times. The area under the ROC curve (AUC) estimates the discrimination ability.

## 3. Results

### 3.1. Patient characteristics

**Figure 1:**
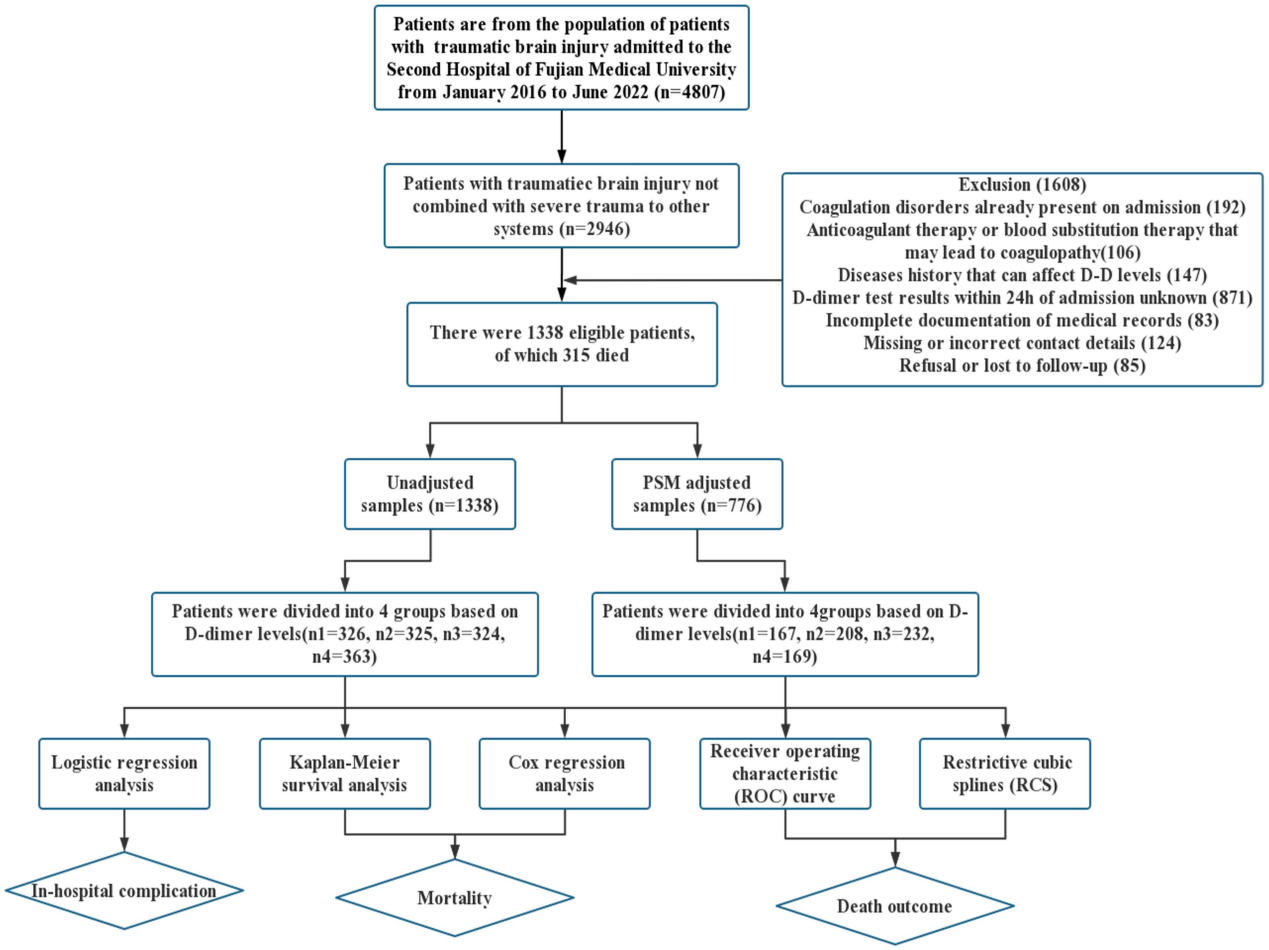
Flow chart of the patient selection process and statistical analysis method.

This cohort study included 1,338 patients with TBI (Figure 1). The baseline characteristics of the patients, stratified by D-dimer levels, are presented in Table 1. The D-dimer levels of the four groups were as follows: G1 (0.00–1.54, *n* = 326), G2 (1.55–6.35, *n* = 325), G3 (6.36–19.99, *n* = 324), and G4 (≥20, *n* = 363). The mean age, GCS, surgical treatment, and laboratory index test results (HB concentration, Plt count, Fib concentration, APTT, TT, and PT) differed between the four groups (*p* < 0.05). In contrast, there were no significant differences in sex and previous medical history (hypertension or diabetes) (*p* > 0.05).

**Table 1:** Baseline patient characteristics stratified by baseline D-dimer levels.

	D-Dimer Groups (mg/L)				
Patient Characteristics	G1^a^	G2^b^	G3^c^	G4^d^	*p-*value for trend
Mean age, years (SD)	45.7 (22.6)	45.5 (22.9)	47.0 (22.0)	52.7 (20.6)	<0.001
Sex					
Male	222 (68.1)	229 (70.5)	207 (63.9)	243 (66.9)	0.347
Female	104 (31.9)	96 (29.5)	117 (36.1)	120 (32.7)	0.347
Medical history					
Hypertension	78 (23.9)	84 (25.8)	66 (20.4)	81 (22.3)	0.393
Diabetes	31 (9.5)	28 (8.6)	29 (9.0)	44 (12.1)	0.392
Glasgow Coma Scale score					
13–15	291 (89.3)	224 (68.9)	183 (56.5)	125 (34.4)	<0.001
9–12	18 (5.5)	47 (14.5)	60 (18.5)	42 (11.6)	
3–8	17 (5.2)	54 (16.6)	81 (25.0)	196 (54.0)	
Surgical treatment					
Yes	83 (25.5)	71 (21.8)	74 (22.8)	178 (49.0)	<0.001
No	243 (74.5)	254 (78.2)	250 (77.2)	185 (51.0)	
Mean baseline biomarker concentrations (SD)					
Hemoglobin, g/L	132.2 (17.3)	128.2 (20.8)	128.1 (22.9)	124.7 (22.6)	<0.001
Platelet, 10^9/L	257.5 (84.2)	243.1 (83.8)	232.1 (82.5)	217.1 (73.3)	<0.001
Fibrinogen, g/L	3.0 (1.2)	2.9 (1.8)	2.6 (1.2)	2.1 (1.1)	<0.001
Activated partial thromboplastin time, s	28.2 (5.4)	27.4 (5.1)	27.4 (6.3)	31.4 (16.9)	<0.001
Thrombin time, s	17.1 (2.8)	16.9 (3.0)	17.2 (2.2)	18.9 (6.2)	<0.001
Prothrombin time, s	13.0 (8.3)	12.5 (1.8)	12.8 (2.4)	13.6 (3.4)	0.017

^a^G1, Group 1: D-dimer level of 10.00–1.54 (*n*_1_ = 326); ^b^G2, Group 2: D-dimer level of 1.55–6.35 (*n*_2_ = 325); ^c^G3, Group 3: D-dimer level of 6.36–19.99 (*n*_3_ = 324); ^d^G4, Group 4: D-dimer level ≥ 20 (*n*_4_ = 363).

### 3.2. Association between D-dimer levels and in-hospital complications

The hospitalization complications are presented in Table 2. We divided the patients into two groups according to a median D-dimer cut-off value of 6.85 mg/L. After a preliminary comparison, we found that high D-dimer levels were associated with serum electrolyte disorders, hypoproteinemia, cardiac complications, urinary system complications, coagulation disorders, pulmonary infection, ICU admission, gastrointestinal complications, liver dysfunction, stress hyperglycemia, deep vein thrombosis, seizures, and intracranial infection during hospitalization (*p* < 0.05). After adjustment by multivariate logistic regression analysis, compared with low D-dimer levels, high D-dimer levels had significant value in predicting serum electrolyte disorders, hypoproteinemia, cardiac complications, urinary complications, coagulation disorders, pulmonary infection, and ICU admission (*p* < 0.05). Correlation analysis after PSM adjustment showed significant differences between high D-dimer levels and serum electrolyte disorder, pulmonary infection, and ICU admission (*p* < 0.05).

**Table 2:** In-hospital complications stratified by low and high D-dimer levels.

Outcomes	Unadjusted		Multivariable Regression Adjustment		Propensity Score Adjustment	
	OR (95% CI)	*p-*value	OR (95% CI)	*p-*value	OR (95% CI)	*p-*value
Serum electrolyte disorder	2.059 (1.603–2.644)	<0.001	1.57 (1.19–2.071)	0.001	1.562 (1.129–2.162)	0.007
Hypoproteinemia	2.162 (1.559–2.998)	<0.001	1.455 (1.005–2.106)	0.047	1.269 (0.826–1.95)	0.276
Cardiac complication	4.44 (1.936–10.181)	<0.001	3.12 (1.307–7.46)	0.01	2.037 (0.813–5.105)	0.129
Urinary complication	3.551 (2.255–5.591)	<0.001	1.743 (1.057–2.874)	0.029	1.569 (0.864–2.848)	0.139
Traumatic coagulopathy	5.007 (2.065–12.141)	<0.001	2.85 (1.098–7.399)	0.031	0.831 (0.252–2.746)	0.14
Pulmonary infection	3.109 (2.477–3.902)	<0.001	1.888 (1.402–2.543)	<0.001	1.362 (1.017–1.824)	0.038
Admission to intensive care unit	6.18 (4.027–9.482)	<0.001	2.495 (1.488–4.185)	0.001	1.926 (1.081–3.43)	0.026
Gastrointestinal complications	2.113 (1.379–3.237)	0.001	1.123 (0.69–1.828)	0.641	0.848 (0.482–1.491)	0.566
Hypohepatia	2.206 (1.591–3.057)	<0.001	1.255 (0.871–1.809)	0.223	1.138 (0.728–1.779)	0.57
Stress hyperglycemia	6.609 (1.486–29.401)	0.013	2.308 (0.487–10.948)	0.292	2.52 (0.486–13.066)	0.271
Deep venous thrombosis	3.711 (1.031–13.363)	0.045	2.485 (0.639–9.667)	0.189	2.01 (0.366–11.041)	0.422
Seizures	1.559 (1.003–2.423)	0.049	1.021 (0.623–1.672)	0.934	1.542 (0.86–2.765)	0.146
Intracranial infection	2.678 (1.178–6.089)	0.019	2.409 (0.882–6.579)	0.086	3.395 (0.927–12.432)	0.065

OR, odds ratio; CI, confidence interval.

### 3.3. Association between D-dimer levels and mortality

**Figure 2:**
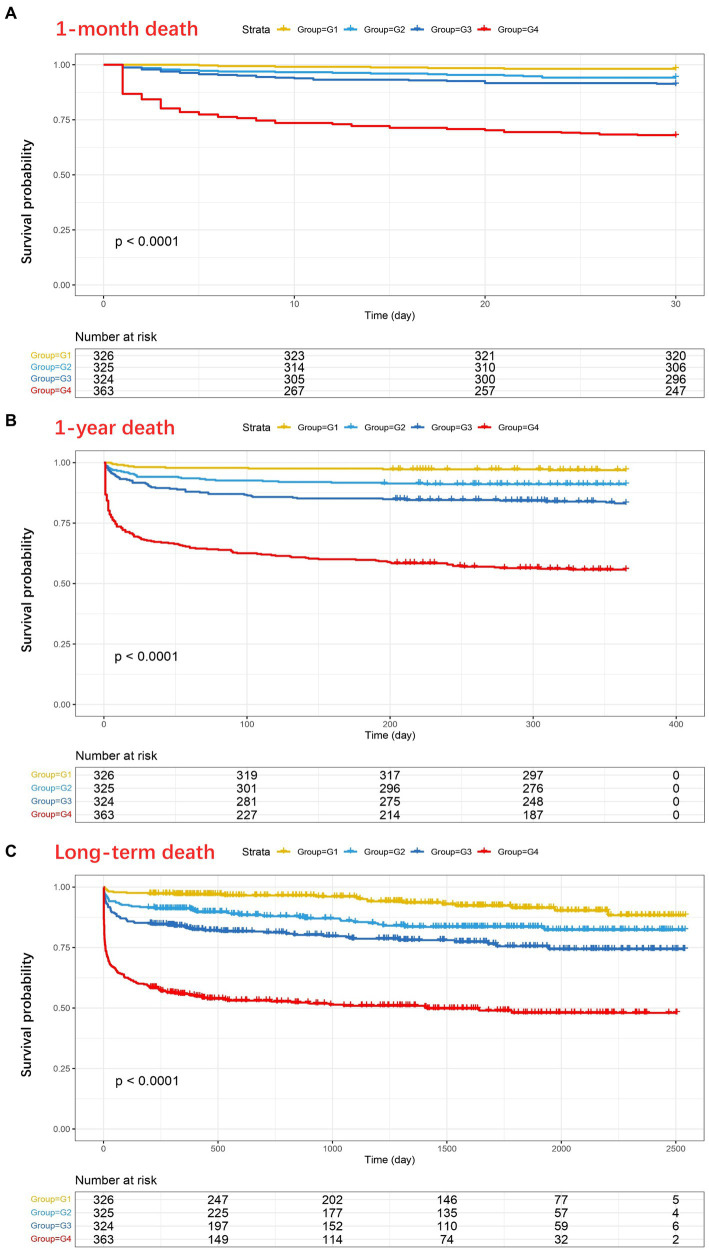
Kaplan–Meier analysis showing the four groups of D-dimer levels in relation to 1-month mortality **(A)**, 1-year mortality **(B)**, and long-term mortality **(C)** after traumatic brain injury (TBI) (*P*_a_ < 0.0001, *P*_b_ < 0.0001, *P*_c_ < 0.0001). As shown in **(C)**, at 2500 days, 5, 4, 6, and 2 patients in the four groups (G1–G4, respectively) did not experience any outcome events and were still being followed up with.

The Kaplan–Meier analysis showed that death events during follow-up were more common in patients with higher D-dimer levels (*p* < 0.001) (Figure 2). The 1-month, 1-year, and long-term survival rates of patients in G4 were significantly lower than those of patients in G1, G2, and G3, and the trend in the survival curve among the four groups was significantly different (*p* < 0.001). A similar trend was observed in the 1-month, 1-year, and long-term survival curves.

Cox regression models were used to further analyze mortality in patients with different D-dimer levels. In univariate analysis, higher D-dimer levels were independently associated with the risk of death, with significantly higher long-term mortality in groups G2, G3, and G4 than in G1 (*p* < 0.001, Table 3). In addition, age, sex, GCS, surgical treatment, HB concentration, Plt count, Fib concentration, APTT, TT, and PT were independent predictors of long-term mortality, and were, therefore, included in the multivariate Cox regression analysis model (Table 4). The results showed that compared to patients in G1 and G2 (aHR 1.655, 95% CI 0.963–2.843), patients in G3 (aHR 2.38, 95% CI 1.416–4.000) and G4 (aHR 3.635, 95% CI 2.195–6.018) had higher long-term mortality (*p* < 0.001; Table 3). After PSM-adjusted analysis, the trend remained significant (*p* < 0.001; Table 3). We found a similar trend in the 1-month and 1-year mortality rates (Tables 3–6).

**Table 3:** Unadjusted and adjusted associations between quartiles of D-dimer levels and mortality.

Outcome	D-Dimer level (mg/L)	No. of deaths (%)	Unadjusted HR (95% CI)	*p*-value	Multivariable regression adjusted HR (95% CI)	*p*-value	PSM-adjusted HR (95% CI)	*p*-value
1-month mortality	0.00–1.54	6 (0.8)	Ref	<0.001	Ref	<0.001	Ref	<0.001
	1.55–6.35	19 (5.8)	3.232 (1.291–8.093)		2.314 (0.822–6.512)		3.302 (1.104–9.878)	
	6.36–19.99	29 (9.0)	5.014 (2.082–12.077)		2.531 (0.926–6.916)		1.629 (0.502–5.288)	
	≥20	117 (32.2)	20.852 (9.178–47.376)		5.413 (2.037–14.386)		7.882 (2.771–22.424)	
1-year mortality	0.00–1.54	10 (3.1)	Ref	<0.001	Ref	<0.001	Ref	<0.001
	1.55–6.35	29 (8.9)	2.998 (1.461–6.152)		2.227 (0.984–5.042)		2.384 (1.008–5.639)	
	6.36–19.99	54 (16.7)	5.788 (2.948–11.366)		3.482 (1.6–7.576)		2.516 (1.084–5.839)	
	≥20	160 (44.1)	18.79 (9.916–35.608)		5.9 (2.747–12.675)		6.413 (2.873–14.317)	
Long-term mortality	0.00–1.54	22 (6.7)	Ref	<0.001	Ref	<0.001	Ref	<0.001
	1.55–6.35	47 (14.5)	2.254 (1.358–3.739)		1.655 (0.963–2.843)		2.34 (1.14–4.803)	
	6.36–19.99	69 (22.3)	3.511 (2.173–5.674)		2.38 (1.416–4)		2.316 (1.14–4.7)	
	≥20	177 (48.8)	10.284 (6.598–16.03)		3.635 (2.195–6.018)		5.023 (2.534–9.955)	

HR, hazard ratio; CI, confidence interval; PSM, propensity score matching.

**Table 4:** Multivariate analysis for long-term mortality.

Characteristics	Unadjusted		Multivariable regression adjustment	
	HR (95% CI)	*p*-value	aHR (95% CI)	*p*-value
Age, year, mean	1.034 (1.027–1.04)	<0.001	1.039 (1.031–1.048)	<0.001
Sex				
Female	1 [Reference]		1 [Reference]	
Male	1.297 (1.013–1.663)	0.039	1.352 (1.036–1.765)	0.027
Medical history				
Hypertension	1.738 (1.375–2.197)	<0.001	1.053 (0.796–1.392)	0.718
Diabetes	1.861 (1.371–2.524)	<0.001	1.108 (0.779–1.578)	0.567
Glasgow Coma Scale score				
13–15	1 [Reference]	<0.001	1 [Reference]	<0.001
9–12	3.802 (2.608–5.544)	<0.001	2.700 (1.817–4.012)	<0.001
3–8	10.991 (8.333–14.496)	<0.001	6.835 (4.886–9.562)	<0.001
Surgical treatment	2.923 (2.342–3.648)	<0.001	1.040 (0.803–1.348)	0.766
Baseline biomarker concentrations				
Hemoglobin, g/L	0.988 (0.983–0.993)	<0.001	0.997 (0.991–1.002)	0.22
Platelet, 10^9/L	0.996 (0.994–0.997)	<0.001	1.001 (1.000–1.003)	0.067
Fibrinogen, g/L	0.866 (0.781–0.96)	0.006	0.990 (0.907–1.080)	0.814
Activated partial thromboplastin time, s	1.025 (1.021–1.03)	<0.001	1.010 (1.003–1.017)	0.004
Thrombin time, s	1.056 (1.041–1.071)	<0.001	1.027(1.005–1.049)	0.014
Prothrombin time, s	1.025 (1.015–1.034)	<0.001	1.021 (1.004–1.039)	0.015
D-Dimer, mg/L				
G1	1 [Reference]	<0.001	1 [Reference]	<0.001
G2	2.254 (1.358–3.739)	0.002	1.655 (0.963–2.843)	0.068
G3	3.511 (2.173–5.674)	<0.001	2.380 (1.416–4.000)	0.001
G4	10.284 (6.598–16.03)	<0.001	3.635 (2.195–6.018)	<0.001

CI, confidence interval; G1, Group 1: D-dimer level of 10.00–1.54 (*n*_1_ = 326); G2, Group 2: D-dimer level of 1.55–6.35 (*n*_2_ = 325); G3, Group 3: D-dimer level of 6.36–19.99 (*n*_3_ = 324); G4, Group 4: D-dimer level ≥ 20 (*n*_4_ = 363).

**Table 5:** Multivariate analysis for 1-month mortality.

Characteristics	Unadjusted		Multivariable regression adjustment	
	HR (95% CI)	*p*-value	aHR (95% CI)	*p*-value
Age, year, mean	1.02 (1.013–1.028)	<0.001	1.021 (1.011–1.031)	<0.001
Sex				
Female	1 [Reference]		1 [Reference]	
Male	1.216 (0.873–1.694)	0.247	–	–
Medical history				
Hypertension	1.146 (0.814–1.615)	0.435	–	–
Diabetes	1.761 (1.167–2.656)	0.007	1.083 (0.676–1.736)	0.74
GCS score				
13–15	1 [Reference]	<0.001	1 [Reference]	<0.001
9–12	7.964 (4.252–14.916)	<0.001	6.28 (3.313–11.902)	<0.001
3–8	23.83 (14.17–40.077)	<0.001	14.619 (8.226–25.982)	<0.001
Surgical treatment	2.76 (2.044–3.727)	<0.001	0.714 (0.509–1.001)	0.051
Baseline biomarker concentrations				
Hemoglobin, g/L	0.989 (0.983–0.996)	0.001	1.001 (0.995–1.008)	0.663
Platelet, 10^9/L	0.995 (0.993–0.997)	<0.001	1 (0.998–1.002)	0.969
Fibrinogen, g/L	0.668 (0.569–0.784)	<0.001	0.94 (0.83–1.064)	0.328
Activated partial thromboplastin time, s	1.025 (1.02–1.03)	<0.001	1.006 (0.999–1.013)	0.1
Thrombin time, s	1.064 (1.05–1.079)	<0.001	1.027 (1.005–1.051)	0.018
Prothrombin time, s	1.03 (1.02–1.039)	<0.001	1.03 (1.012–1.049)	0.001
D-Dimer, mg/L				
G1	1 [Reference]	<0.001	1 [Reference]	<0.001
G2	3.232 (1.291–8.093)	0.012	2.314 (0.822–6.512)	0.112
G3	5.014 (2.082–12.077)	<0.001	2.531 (0.926–6.916)	0.07
G4	20.852 (9.178–47.376)	<0.001	5.413 (2.037–14.386)	0.001

CI, confidence interval; HR, hazard ratio; G1, Group 1: D-dimer level of 10.00–1.54 (*n*_1_ = 326); G2, Group 2: D-dimer level of 1.55–6.35 (*n*_2_ = 325); G3, Group 3: D-dimer level of 6.36–19.99 (*n*_3_ = 324); G4, Group 4: D-dimer level ≥ 20 (*n*_4_ = 363).

**Table 6:** Multivariate analysis for 1-year mortality.

Patient characteristics	Unadjusted		Multivariable regression adjustment	
	HR (95% CI)	*p*-value	aHR (95% CI)	*p*-value
Age, year, mean	1.027 (1.02–1.033)	<0.001	1.028 (1.02–1.036)	<0.001
Sex				
Female	1 [Reference]		1 [Reference]	
Male	1.315 (0.997–1.734)	0.052	–	–
Medical history				
Hypertension	1.312 (0.997–1.726)	0.052	–	–
Diabetes	1.645 (1.161–2.332)	0.005	1 (0.672–1.488)	>0.999
GCS score				
13–15	1 [Reference]	<0.001	1 [Reference]	<0.001
9–12	5.852 (3.649–9.386)	<0.001	4.256 (2.61–6.941)	<0.001
3–8	18.419 (12.704–26.704)	<0.001	10.811 (7.056–16.566)	<0.001
Surgical treatment	2.919 (2.28–3.737)	<0.001	0.885 (0.666–1.174)	0.396
Baseline biomarker concentrations				
Hemoglobin, g/L	0.988 (0.983–0.993)	<0.001	1 (0.995–1.006)	0.982
Platelet, 10^9/L	0.995 (0.994–0.997)	<0.001	1 (0.999–1.002)	0.85
Fibrinogen, g/L	0.811 (0.721–0.913)	0.001	1.007 (0.917–1.106)	0.883
Activated partial thromboplastin time, s	1.026 (1.021–1.03)	<0.001	1.009 (1.002–1.016)	0.011
Thrombin time, s	1.06 (1.046–1.075)	<0.001	1.027 (1.005–1.05)	0.016
Prothrombin time, s	1.028 (1.019–1.037)	<0.001	1.028 (1.011–1.045)	0.001
D-Dimer, mg / L				
G1	1 [Reference]	<0.001	1 [Reference]	<0.001
G2	2.998 (1.461–6.152)	0.003	2.227 (0.984–5.042)	0.055
G3	5.788 (2.948–11.366)	<0.001	3.482 (1.6–7.576)	0.002
G4	18.79 (9.916–35.608)	<0.001	5.9 (2.747–12.675)	<0.001

CI, confidence interval; G1, Group 1: D-dimer level of 10.00–1.54 (*n*_1_ = 326); G2: Group 2: D-dimer level of 1.55–6.35 (*n*_2_ = 325); G3, Group 3: D-dimer level of 6.36–19.99 (*n*_3_ = 324); G4, Group 4: D-dimer level ≥ 20 (*n*_4_ = 363); HR, hazard ratio.

**Figure 3:**
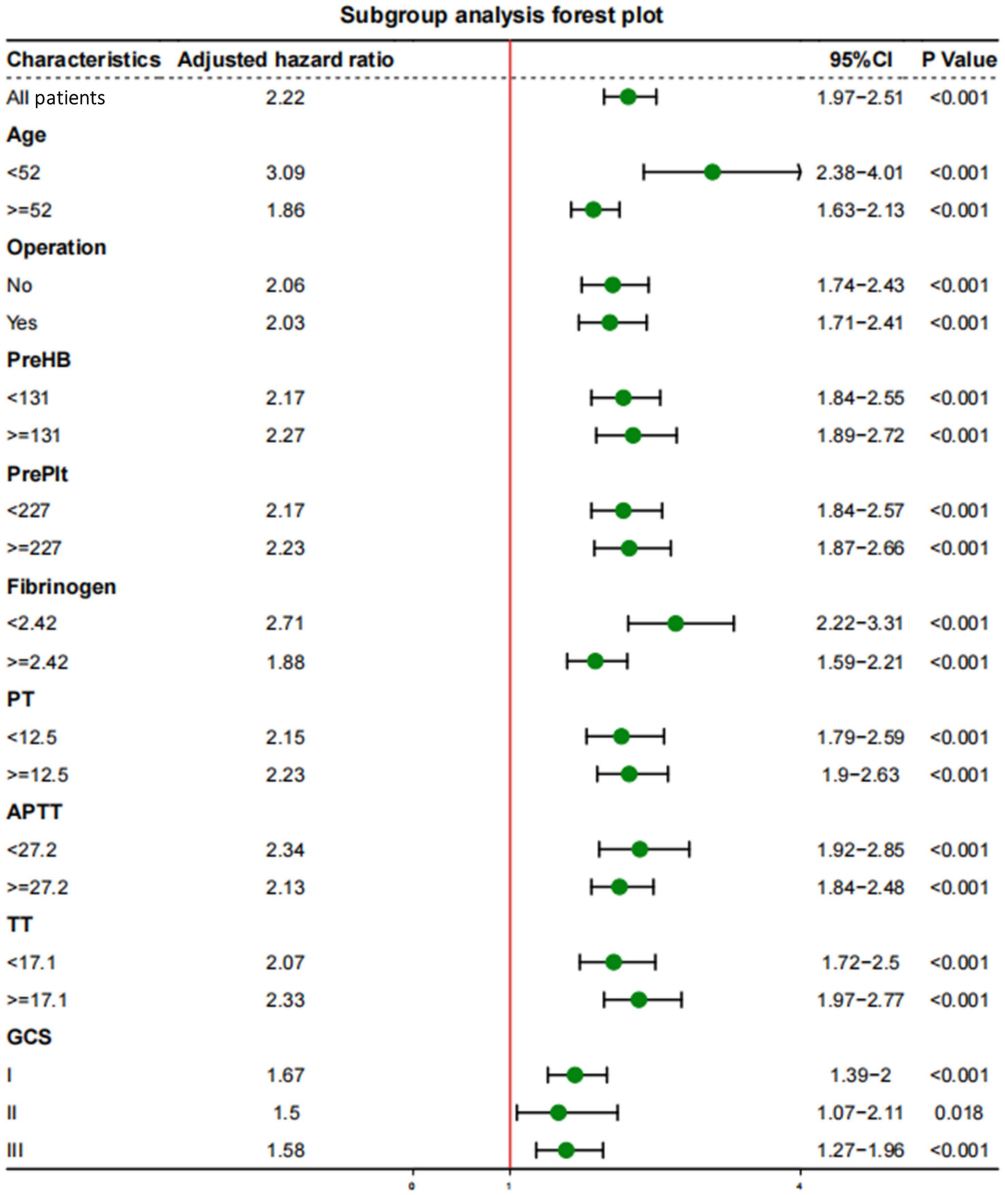
Subgroup analyses of the association between D-dimer levels and long-term mortality with a multivariate model adjusted for age, operation treatment, Glasgow Coma Scale (GCS) score, and concentrations of hemoglobin (HB), platelets (Plt), fibrinogen, prothrombin time (PT), activated partial thromboplastin time (APTT), and thrombin time (TT).

Subgroup analyses were performed to explore whether the interaction of other variables would affect the value of D-dimer levels in predicting mortality outcomes (Figure 3). After setting age, surgical treatment, GCS score, HB concentration, Plt count, fibrinogen concentration, PT, APTT, and TT as separate subgroups, the correlation between D-dimer levels and long-term mortality remained of high predictive value (*p* < 0.001).

### 3.4. Predictive value of D-dimer levels for death outcomes

**Figure 4:**
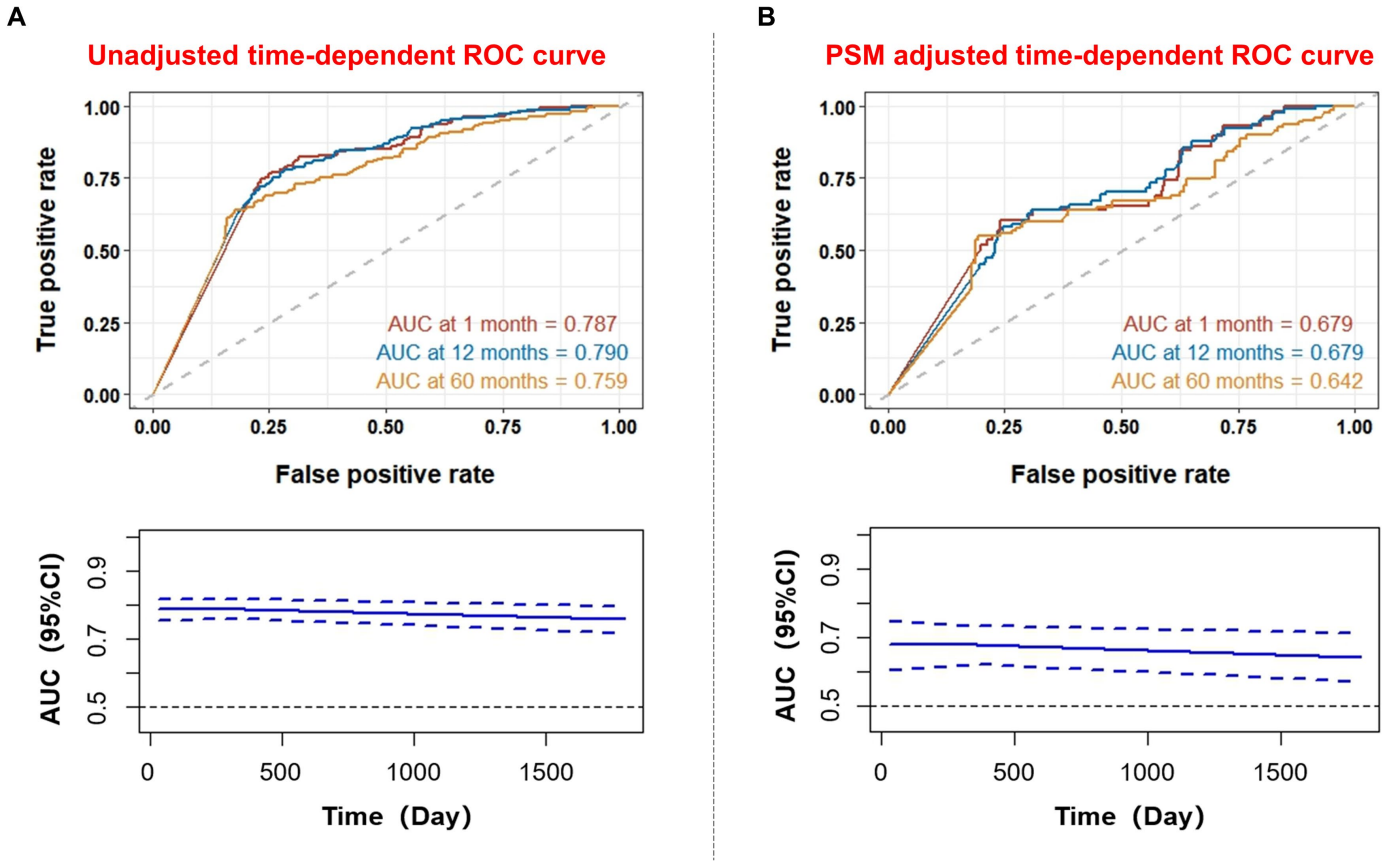
Time-dependent receiver operating characteristic curve for prediction of D-dimer levels in patients with traumatic brain injury. **(A)** Unadjusted time-dependent ROC curve; **(B)** PSM adjusted time-dependent ROC curve. AUC, area under the curve; ROC, receiver operating characteristic; PSM, propensity score matching.

Time-related ROC curves were used to further explore the predictive value of D-dimer levels for the occurrence of death (Figure 4). The results showed that D-dimer levels had different predictive abilities for mortality outcomes at different times. The AUC for long-term mortality was 0.759 (95%CI: 0.720–0.799), while the AUC of 1-year mortality and 1-month mortality was 0.790 (95%CI: 0.761–0.819) and 0.787 (95%CI: 0.755–0.812), respectively. Among them, D-dimer was a long-term prognostic predictor of TBI; the cut-off values were 15.6, 12.53, and 15.95 mg/L for long-term death events, 1-year death events, and 1-month death events, respectively. The best predictive value for the ROC curve was achieved at these cut-off concentrations. After PSM adjustment, the AUC of the ROC curve at different time points was >0.6, indicating predictive significance.

**Figure 5:**
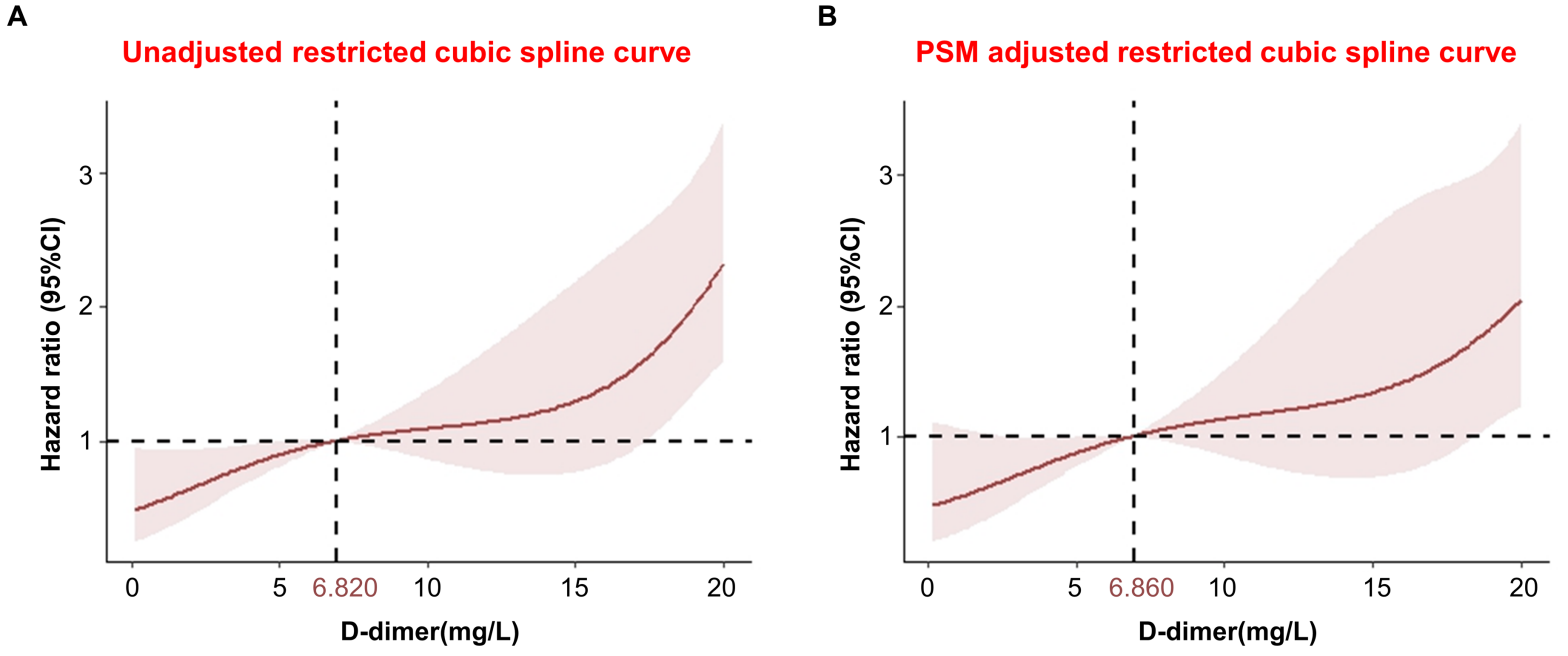
Restricted cubic spline showing the association between D-dimer levels and long-term mortality. **(A)** Unadjusted restricted cubic spline curve; **(B)** PSM adjusted restricted cubic spline curve. CI, confidence interval; PSM, propensity score matching.

In addition, RCS models were used to flexibly and visually predict the relationship between D-dimer levels and long-term mortality in patients with TBI (Figure 5). As shown in Figure 5, the risk of long-term death was relatively low at D-dimer levels <6.820 mg/L. The predicted D-dimer concentration was 6.820 mg/L (6.860 mg/L after PSM adjustment). The risk of death began to increase rapidly, and the HR increased with an increase in D-dimer levels, showing a positive correlation (*p* < 0.001).

## 4. Discussion

TBI is a global health concern due to its association with high mortality rates, disability, and poor prognosis (Wiles et al., 2023). Therefore, early and accurate assessment of injuries is crucial to promptly identify critically ill patients, guide treatment decisions, and establish realistic expectations for the disease. However, the current diagnostic and grading criteria for TBI that are used in clinical practice fail to accurately evaluate all patients (Maas et al., 2022). Therefore, an objective index that can reflect the severity of trauma is urgently required to supplement existing schemes (Maas et al., 2022). The role of D-dimers in the evaluation of TBI has been a topic of interest. It reflects the high fibrinolytic state of the coagulation system and is often used to evaluate venous thrombosis and pulmonary embolism after TBI (Chen et al., 2023). For changes in coagulation function in patients with TBI, D-dimer can respond quickly in the early stages, and changes can be detected in the blood within minutes after TBI (Zhang et al., 2018). Prior studies have shown that D-dimer concentrations in the peripheral blood positively correlate with the risk of progressive hemorrhagic injury in patients with TBI and that D-dimer levels in patients with isolated TBI are associated with poor neurological prognosis 6 months after discharge (Xu et al., 2020; Fujiwara et al., 2022). However, there are no existing studies on the correlation between D-dimer levels and the incidence of complications or long-term mortality during hospitalization in patients with TBI.

Studies have revealed that TBI can have widespread pathophysiological effects on peripheral organs, such as the heart, lungs, gastrointestinal tract, liver, and kidneys, due to autonomic nervous system disorders and systemic inflammatory response (McDonald et al., 2020). By preventing or improving non-neurological complications, such as pneumonia, heart failure, and renal failure, the prognosis of a considerable number of patients can be significantly improved (Robba et al., 2020). In this large single-center cohort study, we found that the D-dimer level at admission was an independent predictor of complications during hospitalization after TBI. High D-dimer levels are associated with serum electrolyte disorders, hypoproteinemia, cardiac complications, urinary complications, coagulation disorders, pulmonary infections, ICU admission, gastrointestinal complications, liver dysfunction, stress hyperglycemia, deep vein thrombosis, epilepsy, and intracranial infections during hospitalization. In the present study, serum electrolyte disorder, pulmonary infection, and admission to the ICU remained significant after multivariate adjustment and PSM adjustment of the sample data and have been reported to be significantly associated with high mortality in patients with TBI (Longhi et al., 2017; Sundman et al., 2017; Sharma et al., 2019; Tsai et al., 2020).

In our study, the D-dimer level was identified as an independent predictor of short- and long-term mortality after TBI. Several small studies have assessed the short-term mortality and poor prognosis in patients with TBI. For example, studies have found that higher levels of D-dimer are associated with higher in-hospital, 28-day (Allard et al., 2009; Lee et al., 2018), and 30-day mortality (Yuan et al., 2012). Fujiwara et al. analyzed a multicenter TBI database and found that D-dimer levels at admission were associated with poor GOS scores and mortality at 6 months (Fujiwara et al., 2022). Our 1-month and 1-year mortality results are similar to these findings, indicating that D-dimer levels are associated with short-term mortality in patients with TBI, which further supports the credibility of our study. However, to the best of our knowledge, previous studies have not discussed the relationship between long-term mortality and poor prognosis with D-dimer levels in patients with TBI. In the present study, through long-term follow-up observations, a large sample of prognostic data was collected over a long duration. The average follow-up time was 2.8 years, and the longest follow-up time was 6.9 years. Relatively complete prognostic data were available, which could be used to determine the correlation between D-dimer levels and long-term mortality of patients with TBI and provide further evidence supporting the role of D-dimer levels in the prediction of poor prognosis. We found a possible dose–response relationship between elevated D-dimer levels and patient mortality. The odds of mortality were significantly higher in patients with higher D-dimer levels than in patients with lower D-dimer levels.

In addition, in most previous studies, the methods used to determine the correlation between D-dimer levels and TBI were relatively simple. For example, Simone et al. found that the D-dimer level can be used as an evaluation method to reduce the use of unnecessary CT examinations in children with TBI through ROC model analysis (Langness et al., 2018), and Xu et al. (2020) found that the ratio of D-dimer/Fib can predict the incidence of progressive hemorrhagic injury after TBI through logistic regression and the ROC model. In this study, various statistical methods and models were used to determine the relationship between D-dimer levels and patient mortality at different time points. These methods included Kaplan–Meier survival analysis, Cox multivariate regression, an ROC model, RCS model, and subgroup analysis. The use of these statistical approaches and models enhanced the rigor of the study’s statistical methods and design. Furthermore, previous studies failed to account for confounding bias. In our study, we addressed the issue by excluding confounding factors such as age, GCS score, surgery, and other blood test indicators. This allowed us to determine whether the D-dimer level was an independent predictor of death in patients with TBI. The dataset was adjusted using PSM to verify the reliability of the results. After adjusting for confounding factors and performing subgroup analysis, our findings remained significant, which is one of the advantages of our study.

There is no direct pathological evidence indicating the association between D-dimer levels and death in patients with TBI. Based on a literature review, we speculate that the following mechanisms may be involved. First, the D-dimer levels may indicate the severity of tissue damage. In mild TBI, elevated D-dimer levels on admission are independently associated with intracranial structural disorders (Sugimoto et al., 2017); in severe TBI, acute D-dimer levels predict hematoma expansion (Suehiro et al., 2014), and hyperfibrinolysis is more damaging than low fibrinolysis (Hayakawa et al., 2017). D-dimer levels also predict the occurrence of progressive hemorrhagic brain injury after TBI (Xu et al., 2020). In addition, D-dimer regulates the local immune response, stimulates the synthesis of monocytes, and releases pro-inflammatory cytokines, such as interleukin-6, which may contribute to diseased tissue edema and hematoma formation in various diseases (Welch et al., 2016). Elevated D-dimer levels are also associated with intracranial lesions and poor prognosis in patients with cerebral infarction and subarachnoid hemorrhage (Fang et al., 2022; Qiu et al., 2023). Thus, D-dimer levels may indicate brain tissue damage and prognosis. Second, local brain lesions after TBI can alter body coagulation, thereby increasing D-dimer levels. In the acute phase of TBI, tissue factors are released from the damaged blood–brain barrier into the entire body, rapidly increasing plasma D-dimer levels (Kwaan, 2020). Tissue factors promoting fibrinolysis and prothrombin conversion (Suehiro et al., 2019). Thrombin degrades fibrin into the D-dimer and fibrin degradation products. Thrombin-related proteins cause post-traumatic immunosuppression, inflammation, and breakdown of the blood–brain barrier. Low tissue blood perfusion after TBI worsens the imbalance of these coagulation and fibrinolysis systems, causing brain tissue damage and post-traumatic changes in other organs (Kwaan, 2020; Medcalf et al., 2020), resulting in poor prognosis.

This study has several limitations that should be taken into consideration when interpreting our results. First, the retrospective study design limited our ability to establish causality and was susceptible to bias from unmeasured factors. Second, we only assessed one biomarker; however, additional markers may be helpful for risk classification (Böhm et al., 2022). Third, our investigation solely focused on all-cause mortality and did not explore the association between D-dimer levels and cause-specific mortality. According to a previous study, D-dimer levels can influence cardiovascular, cancerous, and non-cardiovascular non-cancerous mortality. Therefore, the association between D-dimer levels and cause-specific mortality should be investigated in future studies(Simes et al., 2018). Fourth, for the long-term prognosis of patients with TBI, we only evaluated adverse outcomes such as death, and there was a lack of recording and analysis of other adverse prognostic states such as paralysis, loss of motor and sensory function, and cognitive dysfunction, and the Glasgow Outcome Scale (Asami et al., 2022) scores were not recorded. Fifth, differences in the time of blood collection and facilities may have resulted in variations in D-dimer levels, which may affect the accuracy of our findings (Asami et al., 2022). In the future, we hope to obtain more accurate and comprehensive follow-up data by implementing a prospective, multicenter, and large-sample design to optimize our research.

In summary, based on the predictive value of D-dimer levels for complications and short- and long-term mortality in patients with TBI during hospitalization, we believe that D-dimer detection is a promising approach for the early assessment of all patients with TBI. As a blood test index, D-dimer can be measured in the acute phase of TBI rapidly and at a low cost. It not only facilitates the prompt identification and diagnosis of patients with different severities of injury but also potentially guides early clinical treatment and long-term rehabilitation measures for patients with TBI (Maegele et al., 2017). The D-dimer test can reduce the need for sophisticated testing tools such as CT and MRI in patients with mild TBI, which will lower treatment costs and radiation exposure. Furthermore, it enables early intervention and the use of targeted measures to improve outcomes in patients with moderate to severe TBI (Foaud et al., 2014). This development is expected to substantially impact how patients with TBI are assessed and treated.

## 5. Conclusion

**Figure 6:**
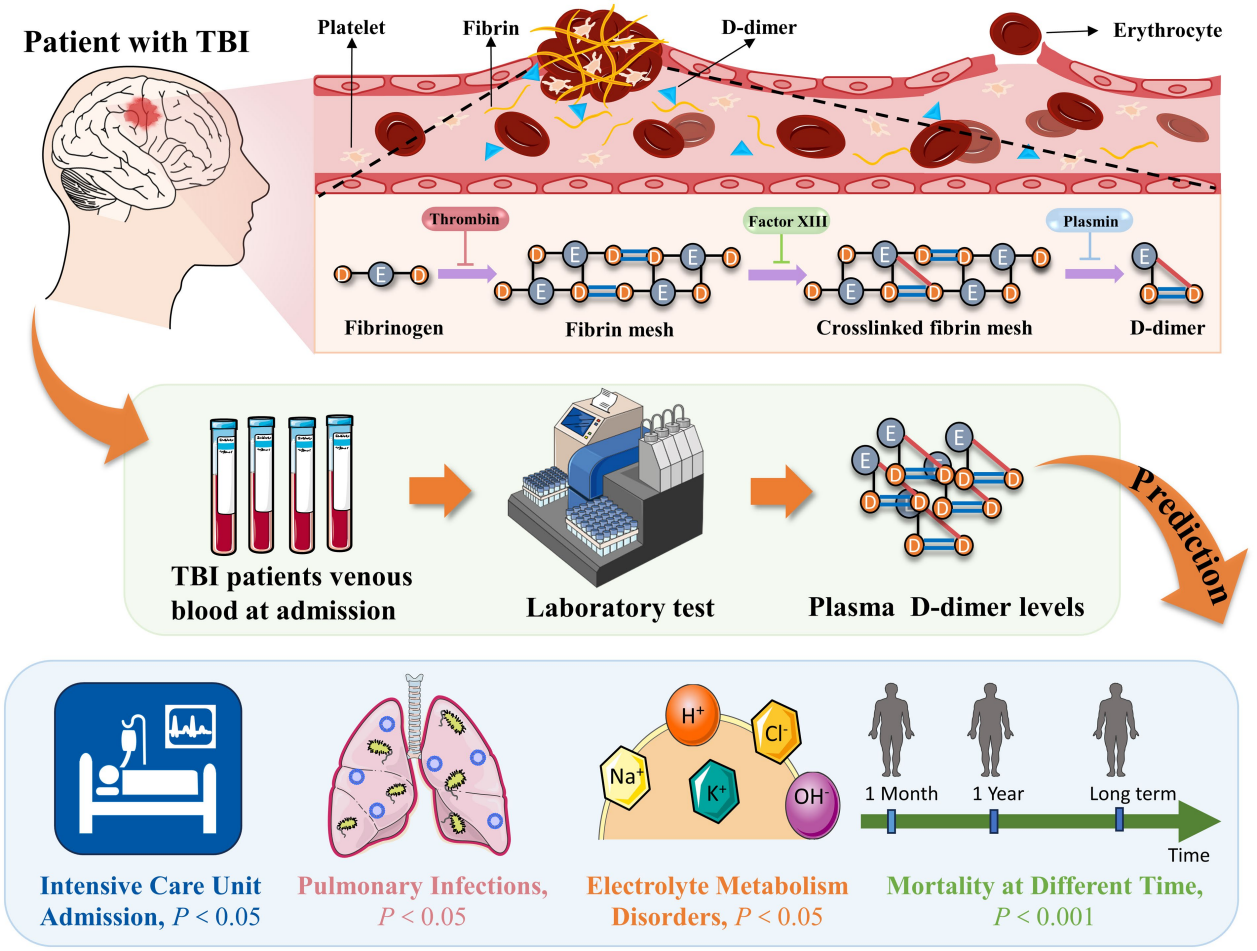
High D-dimer levels within 24 h of admission were associated with complications and mortality in TBI patients. TBI, traumatic brain injury.

In conclusion, our results revealed that in patients with TBI, high D-dimer levels within 24 h of admission were associated with complications such as electrolyte metabolism disorders, pulmonary infections, and ICU admission during hospitalization (*p* < 0.05) (Figure 6). Additionally, we observed a dose–response relationship between elevated D-dimer levels and patient mortality. The odds of mortality were significantly higher in patients with higher D-dimer levels than in those with lower D-dimer levels (*p* < 0.001) (Figure 6). In the future, this blood marker may help to timely identify patients at risk of poor prognosis and tailor treatment plans for these patients, thereby reducing mortality and improving prognosis. Furthermore, our findings may also facilitate the exploration of new therapeutic targets in the future. However, further studies are required to validate our results and elucidate the underlying mechanisms between the correlation of D-dimer levels and clinical outcomes.

## Data availability statement

The datasets presented in this article are not readily available due to privacy or ethical restrictions. Requests to access the datasets should be directed to the corresponding author.

## Ethics statement

The studies involving humans were approved by the Ethics Committee of the Second Affiliated Hospital of Fujian Medical University. The studies were conducted in accordance with the local legislation and institutional requirements. Written informed consent for participation in this study was provided by the participants’ legal guardians/next of kin.

## Author contributions

XC: Conceptualization, Formal analysis, Validation, Writing – original draft, Methodology. XW: Data curation, Investigation, Writing – original draft. YL: Data curation, Investigation, Writing – original draft. XG: Data curation, Investigation, Writing – original draft. FW: Project administration, Writing – original draft. YY: Project administration, Writing – original draft. WH: Supervision, Writing – review & editing. FZ: Supervision, Writing – review & editing. HH: Conceptualization, Funding acquisition, Writing – original draft, Supervision, Writing – review & editing.
